# 2D/2D Heterojunction of R-scheme Ti_3_C_2_ MXene/MoS_2_ Nanosheets for Enhanced Photocatalytic Performance

**DOI:** 10.1186/s11671-020-03314-z

**Published:** 2020-04-09

**Authors:** Ziyu Yao, Huajun Sun, Huiting Sui, Xiaofang Liu

**Affiliations:** 1 State Key Laboratory of Silicate Materials for Architectures, Wuhan University of Technology, Wuhan, 430070 People’s Republic of China; 2 School of Materials Science and Engineering, Wuhan University of Technology, Wuhan, 430070 People’s Republic of China; 3 Advanced Ceramics Institute of Zibo New & High-Tech Industrial Development Zone, Zibo, 255000 People’s Republic of China; 4 School of Chemistry, Chemical Engineering and Life Sciences, Wuhan University of Technology, Wuhan, 430070 People’s Republic of China

**Keywords:** 2D/2D heterojunction, Hydrothermal reaction, Photocatalytic degradation, H_2_ evolution reaction

## Abstract

Combination of two-dimensional (2D) materials and semiconductors is considered to be an effective way for fabricating photocatalysts for solving the environmental pollution and energy crisis. In this work, novel 2D/2D heterojunction of R-scheme Ti_3_C_2_ MXene/MoS_2_ nanosheets is successfully synthesized by hydrothermal reaction. The photocatalytic activity of the Ti_3_C_2_ MXene/MoS_2_ composites is evaluated by photocatalytic degradation and hydrogen evolution reaction. Especially, 0.5 wt% Ti_3_C_2_ MXene/MoS_2_ sample exhibits optimum methyl orange (MO) degradation and H_2_ evolution rate of 97.4% and H_2_ evolution rate of 380.2 μmol h^−1^ g^−1^, respectively, which is attributed to the enhanced optical absorption ability and increased specific surface area. Additionally, Ti_3_C_2_ MXene coupled with MoS_2_ nanosheets is favorable for improving the photocurrent response and reducing the electrochemical impedance, leading to the enhanced electron transfer of excited semiconductor and inhibition of charge recombination. This work demonstrates that Ti_3_C_2_ MXene could be a promising carrier to construct 2D/2D heterojunction in photocatalytic degradation and hydrogen evolution reaction.

## Introduction

Over the past few years, the Industrial Science and Technology is developing significantly, whereas the environmental problems and energy crisis have become much more serious [1–4]. Significant application of titanium oxide (TiO_2_) for splitting water has been reported since 1972 [5]. Researchers have been working to extend the response of the TiO_2_-based composites to visible light region and explore the narrow bandgaps semiconductor to deal with environmental pollution and energy crisis better [6–12].

Metal sulfide semiconductor catalysts have been considered as essential carriers to solve environmental pollution and energy crisis due to the narrow bandgaps, low toxicity and excellent catalytic ability [13, 14]. The relatively narrow bandgap (Eg = 1.8 eV), unique optical properties and layered structure of MoS_2_ nanosheets have attracted more and more attention [15–18]. MoS_2_ has been coupled with several two-dimensional (2D) materials and semiconductors, such as TiO_2_ [19], graphene oxide (GO) [20], g-C_3_N_4_ [21], SnO_2_ [12], Bi_2_WO_6_ [22], Bi_2_O_2_CO_3_ [23], and CdS [24], in order to improve the efficiency of photocatalytic degradation and hydrogen production. It has been proved that higher concentration of methyl orange (MO) (30 mg/L) organic pollutants can be degraded in 60 min under the visible light irradiation by MoS_2_/CdS nanocomposites [24].

Since the initial report in 2011, MXenes, as a member of the two-dimensional material family, has attracted extensive attention of researchers [25–27]. MXenes can be prepared from MAX phase by etching the A-layer with HF or HCl/LiF, which possesses excellent electrochemical properties, chemical stability, and numerous hydrophilic functionalities on the surface (-OH/-O) [28–30]. The most popular Ti_3_C_2_ MXene can be obtained by exfoliating Ti_3_AlC_2_ with strong acid [31]. Its outstanding conductivity and two-dimensional layered structure have been considered as energy storage materials for sodium-ion batteries (SIBs) and electrochemical capacitors [31–34].

Ti_3_C_2_ MXene with rich oxidized surface groups favors the heterojunction formed between MXene and semiconductors [35–38]. The heterojunction assists to establish strong interface contact between photocatalyst and cocatalyst. Due to the strong physical and electronic coupling effect, the interface contact can greatly enhance the transfer and separation of photo-induced carriers on the heterojunction interface, which is the key factor to improve the photocatalytic performance [39–41].

For example, TiO_2_/Ti_3_C_2_ and Ti_3_C_2_/Bi_2_WO_6_ composites have exhibited excellent photocatalytic CO_2_ reduction activity, which is ascribed to the highly efficient charge-carrier separation and rich activation sites [42, 43]. The hydrogen production performance of the g-C_3_N_4_/Ti_3_C_2_ photocatalyst has enhanced significantly, which is attributed to the superior electrical conductivity and highly efficient charge transfer [44]. TiO_2_/Ti_3_C_2_ and α-Fe_2_O_3_/Ti_3_C_2_ hybrids are proved to promote the photocatalytic degradation efficiency of organic pollutants under ultraviolet light and visible light by constructing heterojunctions [45–47].

Herein, 2D/2D heterojunction of R-scheme Ti_3_C_2_ MXene/MoS_2_ photocatalysts is synthesized by hydrothermal method. Photocatalytic activities of Ti_3_C_2_ MXene/MoS_2_ composites are evaluated by photocatalytic degradation of MO and hydrogen evolution reaction (HER) under visible light irradiation. Photocatalytic performance reflects that MoS_2_ coupled with Ti_3_C_2_ MXene presents higher degradation ability and H_2_ production rate than pure MoS_2_ under the same condition. The enlarged specific surface area and enhanced optical absorption ability can be attributed to the morphology of MoS_2_ nanosheets change from crouching to stretching, which is induced by Ti_3_C_2_ MXene. Above all, the strong interaction between MoS_2_ and Ti_3_C_2_ MXene is beneficial to construct 2D/2D heterojunction, which effectively promotes the separation and transfer of photoelectrons from vacancies, thus enhancing the photocatalytic activity significantly.

## Method/Experimental Section

### Photocatalysts Preparation

#### Raw Materials

Ti_3_AlC_2_ MAX powders (> 98 wt% purity), hydrofluoric acid, ammonium molybdate ((NH_4_)_6_Mo_7_O_24_•4H_2_O), thiourea ((NH_2_)_2_CS) and methylene orange are purchased by Shanghai Yuehuan Co., Ltd. (Shanghai, China) and Guoyao Chemical Co., Ltd. (China), respectively.

#### Synthesis of Ti_3_C_2_ Nanosheets

Ti_3_AlC_2_ black powder is etched in 49% HF solutions at room temperature via stirring for 26 h to remove the Al layer. The disposed powder is washed by deionized water via centrifugation 7~8 times until the pH reaches 7. The suspension of Ti_3_C_2_ is sonicated for 6 h and then centrifuged for 20 min at 10,000 rpm [48]. Finally, the solution is dried to obtain the final product Ti_3_C_2_ MXene nanosheets.

#### Hydrothermal Preparation of Ti_3_C_2_ MXene/MoS_2_ (Denoted as TM) Composites

Firstly, 1.1 g of ammonium molybdate ((NH_4_)_6_Mo_7_O_24_•4H_2_O) and 2.2 g of thiourea ((NH_2_)_2_CS) are dissolved in deionized water under vigorous stirring for 60 min to form a homogeneous solution, which is labeled as solution A. Then, an amount of Ti_3_C_2_ nanosheets is added to 20 ml deionized (DI) water stirring for 30 min followed by additional ultrasonication for 40 min, which is labeled as solution B. Then B is mixed into A drop by drop under ultrasonication for 30 min. The mixed solution is transferred into a 100 mL Teflon-lined autoclave and held at 180 °C for 7 h. After cooling to room temperature, the obtained black catalysts are washed by DI water for three times to remove dispersing agent, and then dried at 70 °C for 10 h in a vacuum oven. By adding the Ti_3_C_2_ solution, the mass ratio of Ti_3_C_2_ MXene to MoS_2_ is set as 0, 0.1%, 0.3%, 0.5%, 1.0%, and 2.0 wt%, respectively. The prepared samples are labeled as TM0, TM0.1, TM0.3, TM0.5, TM1, and TM2, respectively.

#### Photocatalytic Degradation of Methylene Orange

All the degradation experiments are carried out in a 100 mL beaker with a constant stirring. Methyl orange is selected to evaluate the photocatalytic activity of the samples. The photocatalytic degradation test of MO is performed by using a 400 W metal halide lamp. In a typical experiment of MO degradation, 50 mg of Ti_3_C_2_/MoS_2_ sample is dispersed into 50 mL MO aqueous solution (20/30/50 mg/L). Then, the solution with catalysts is placed in the dark for 60 min under strong magnetic stirring to establish adsorption equilibrium. The samples are processed by ultrasonic for 1 min before turn on the light, which makes the catalyst dispersed well in the solution. At certain time intervals, approximately 3.5 mL of mixed solution is extracted with centrifugation treatment for 4 min at 8000 rpm^−1^ to remove the solid catalyst powder. The change at 464 nm wavelength is determined by the concentration of the MO solution, which is measured by using an UV-visible spectrophotometer. The initial concentration of the MO solution is labelled as C_0_, and C_t_ refers to the concentration of MO solution at a certain time, respectively. The degradation efficiency of the sample is reflected by the relative absorbance C_t_/C_0_.

#### Photocatalytic Hydrogen Production Evaluation

The photocatalytic H_2_ evolution tests are carried out in a 50 mL quartz flask under ambient temperature and atmospheric pressure. Five milligram of TM sample is dispersed in 70 mL aqueous solution containing 0.35 M Na_2_S and 0.25 M Na_2_SO_3_, and irradiated by 300 W Xe lamp equipped with a 420 nm cutoff filter. Before irradiation, gas (N_2_) is continuously passed through for 35 min to remove the oxygen. The production of H_2_ is detected by gas chromatography (Agilent 7890) equipped with TCD detector.

#### Microstructure Characterization

The phase analysis of the Ti_3_C_2_/MoS_2_ samples is operated at 40 kV and 40 mA by X-ray diffractometer (XRD, Cu Kα, Bruker D8 Advance, Germany). The micro-morphology of the composites is observed by field emission scanning electron microscopy (FESEM, Zeiss Ultra Plus, Zeiss, Germany) coupled with energy-dispersive spectrometry (EDS). High resolution transmission electron microscopy (HRTEM, JEM-2100F, Japanese electronics, China) is used to observe the morphology and heterojunction interface between MoS_2_ and Ti_3_C_2_. The infrared spectra are recorded by Fourier transform infrared spectroscopy (FTIR, Nexus, Therno Nicolet, USA) in a range of 400 to 4000 cm^−1^. The optical properties of powders are performed by UV-Vis diffuse reflectance spectroscope (DRS, Lambda 750S, PerkinElmer, USA) with an integrated sphere. Chemical states of the obtained catalysts are studied by X-ray photoelectron spectroscopy (XPS, ESCALAB 250Xi, Thermo Fisher Scientific, China).

#### Electrochemical Measurements

The electrochemical tests are measured by 1030 A CHI electrochemical station. In a typical experiment, 5 mg of TM sample and 110 μL of 5 wt% Nafion solution are dispersed in 2.5 mL of 1:4 v/v ethanol and water with 9 min sonication to form homogeneous suspension. Subsequently, 5 μL of the ink is dropped onto the glassy carbon electrode (GCE) surface. The electrochemical impedance spectroscopy (EIS) tests are carried out in the same configuration at overpotential *n* = 200 mV from 0.1 to 105 kHz with an AC voltage of 5 mV.

## Results and Discussion

**Fig. 1:**
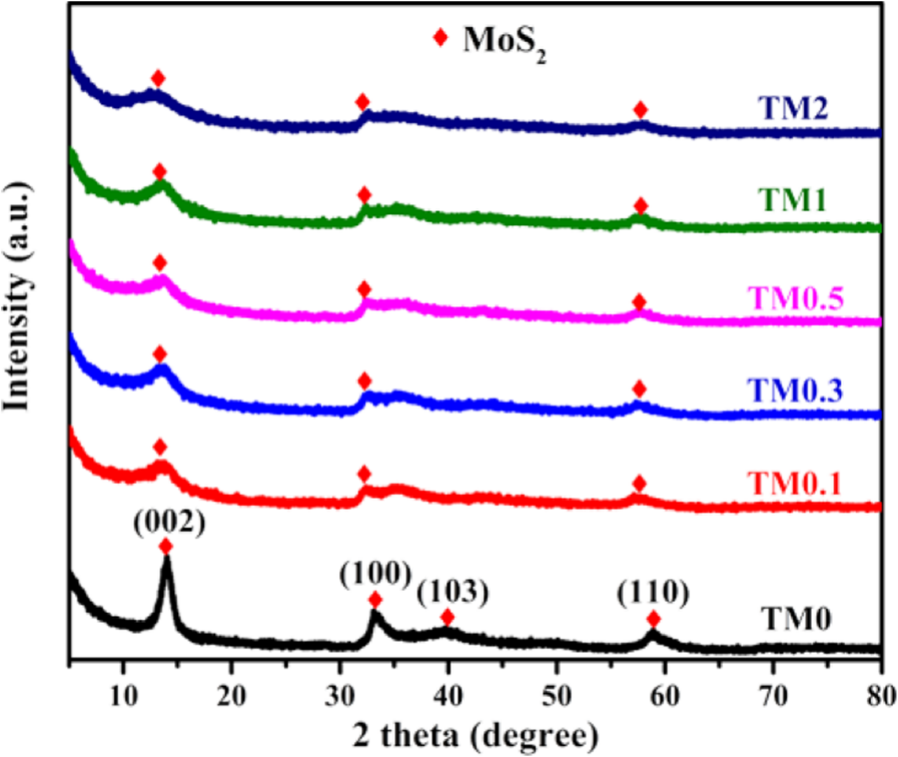
XRD patterns of TM0, TM0.1, TM0.3, TM0.5, TM1, and TM2 composites

Crystalline of Ti_3_AlC_2_ and Ti_3_C_2_ MXene is analyzed in the range of 2*θ* = 5 − 70°, as shown in Fig. S1. The remarkable diffraction peak of Ti_3_AlC_2_ located at 2*θ* = 39° disappears and peak of Ti_3_C_2_ MXene 2*θ* = 9.7° shifts to lower angles, suggesting that Ti_3_AlC_2_ has transformed to Ti_3_C_2_ successfully [42]. Figure 1 reveals XRD patterns of TM samples with various Ti_3_C_2_ additions and the main diffraction peaks of TM0 sample have been indexed to pure MoS_2_ with lattice constants *a =* 3.16 and *c =* 12.294 Å (JCPDS no. 37-1492), respectively [15]. After coupled with Ti_3_C_2_, the main diffraction peaks for (002), (100), and (103) planes of TM composites display broader and decreased intensity than TM0, suggesting that MoS_2_ is suppressed by Ti_3_C_2_ growth limiting effect [49]. No obvious diffraction peak of Ti_3_C_2_ MXene can be detected, which is attributed to the low Ti_3_C_2_ loading with well dispersion in the composites.

**Fig. 2:**
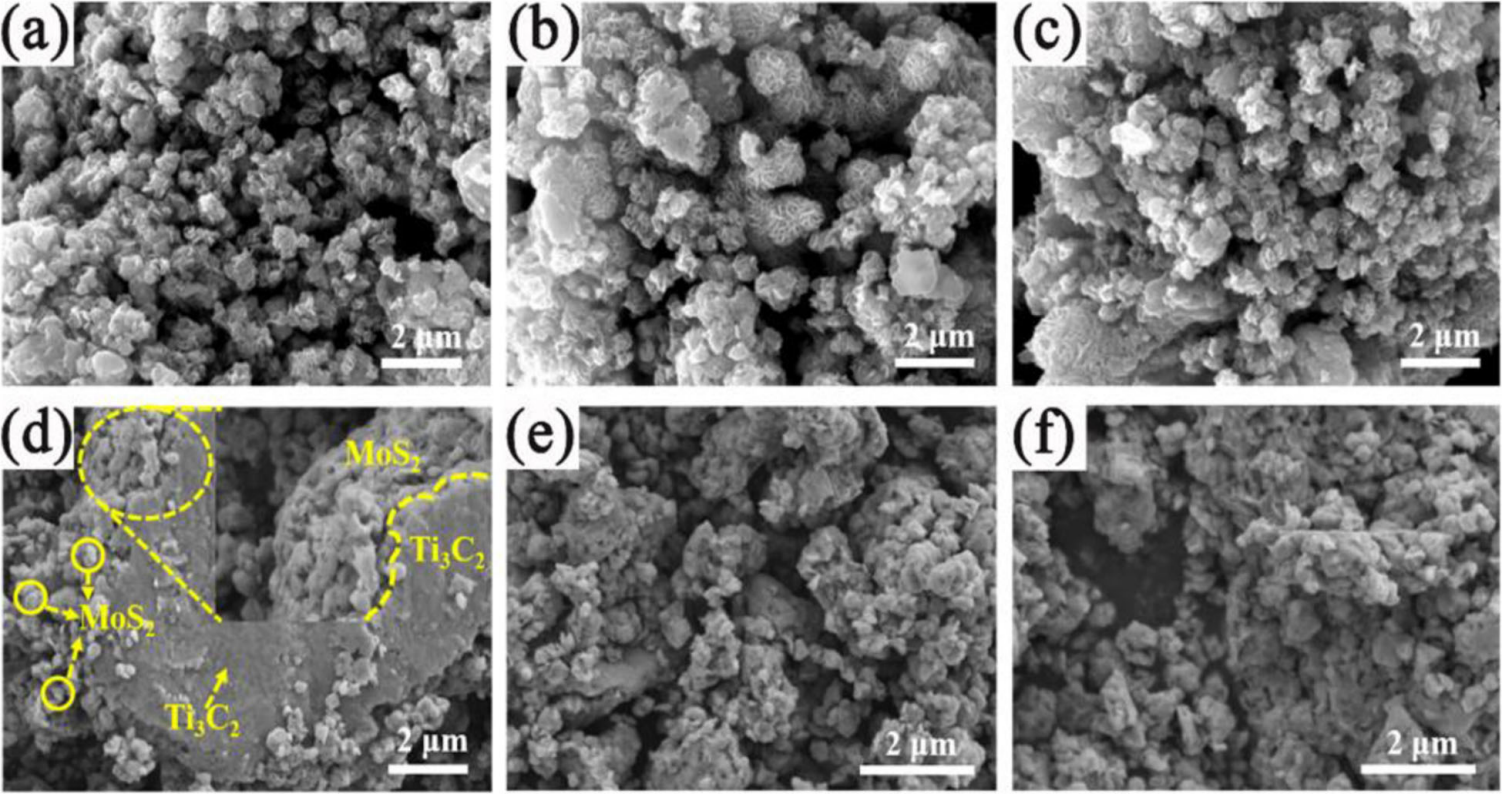
FESEM images of **a** TM0, **b** TM0.1, **c** TM0.3, **d** TM0.5, **e** TM1, and **f** TM2

Morphological images of Ti_3_C_2_/MoS_2_ composite with various Ti_3_C_2_ amounts are observed in Fig. 2. It shows that all of the samples reveal flower-like nanosphere feature with holes separated randomly in the surface. And the flower-like structure of TM composites is composed from irregular nanosheets with average thickness of about 15 nm.

Figure 2a exhibits typical microscopic structure of TM0 with diameter of about 200-400 nm. Figure 2b-f gives FESEM images of TM0.1, TM0.3, TM0.5, TM1, and TM2. It can be seen that all the samples share similar morphology feather with pure MoS_2_. Layered Ti_3_C_2_ MXene has smoother surface and the flower-like MoS_2_ microsphere enrichment at the edge of the lamellae, indicating that the structure of Ti_3_C_2_ MXene is not destroyed during hydrothermal synthesis. Figure S2a reveals the 2D/2D heterojunction with intimate coupling between (2D) MoS_2_ and (2D) Ti_3_C_2_. The corresponding EDS mapping images are obtained in Fig. S2b-e, which reflects that Mo, Ti, and C elements dispersed uniformly in the TM composite.

**Fig. 3:**
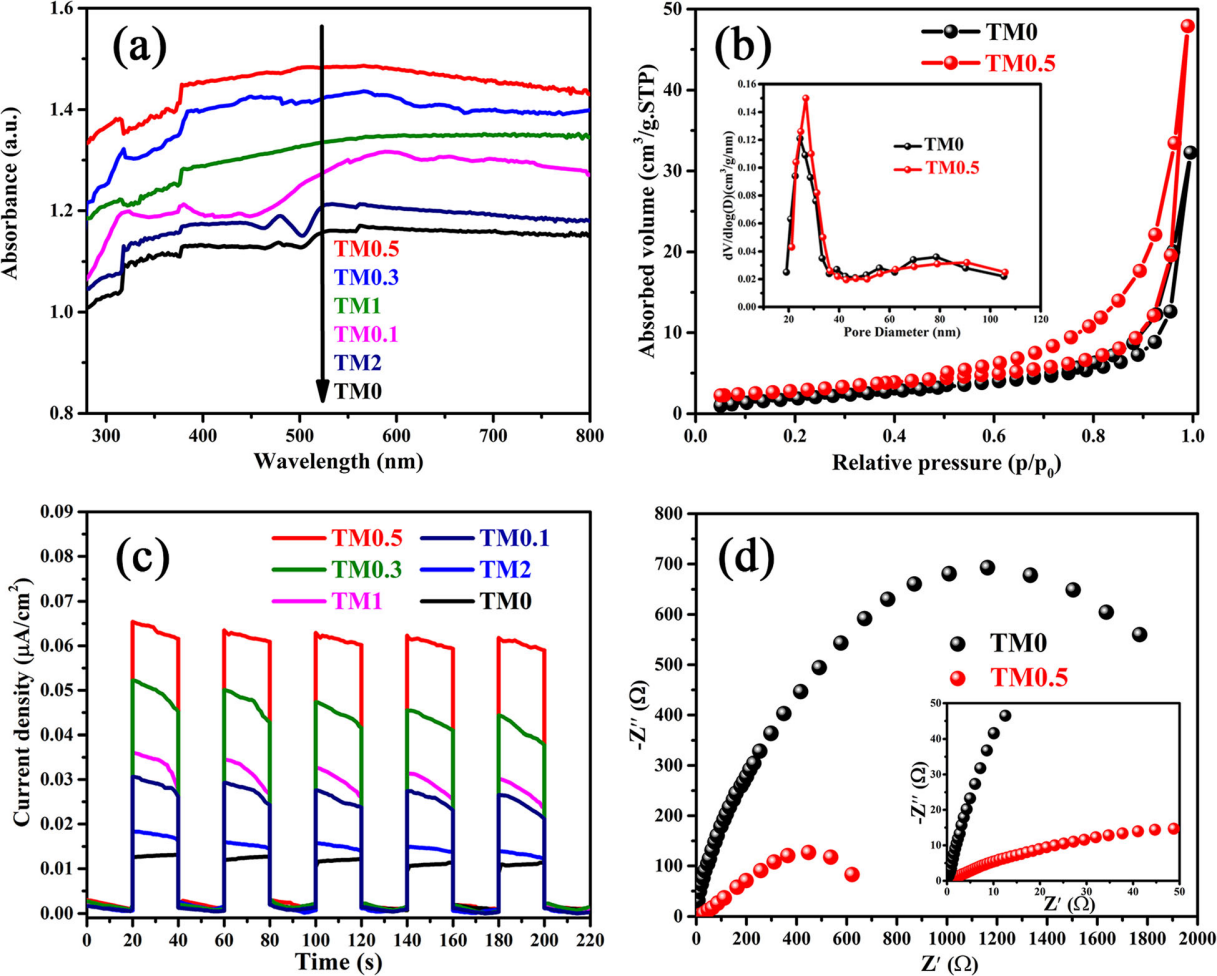
**a** UV-vis diffuse reflectance spectra (DRS) of as-synthesized TM0, TM0.1, TM0.3, TM0.5, TM1, and TM2 samples. **b** N_2_ adsorption-desorption isotherms for the as-prepared TM0 and TM0.5 powders. **c** Photocurrent response of TM0, TM0.1, TM0.3, TM0.5, TM1, and TM2. **d** Electrochemical impedance spectra of TM0 and TM0.5 sample

The optical absorption property of TM composites is analyzed by UV-Vis DRS spectrum, as revealed in Fig. 3a. TM0.5 possesses the strongest optical absorption ability in the range of visible and UV light in sharp contrast with TM0. One can note that in a certain range, the optical absorption intensity of TM composites is enhanced significantly with the increase of Ti_3_C_2_ content. Especially, excessive Ti_3_C_2_ reduces the photocatalytic performance of the TM samples, which is ascribed to the fact that excessive Ti_3_C_2_ addition prevents the light absorption of MoS_2_ nanosheets [50].

Figure 3b shows the N_2_ adsorption-desorption isotherms of TM0 and TM0.5 samples and their pore size distribution curves (Fig. 3b inset). Both of the samples are treated at 100 °C for 4 h before testing. The average pore size of TM0 and TM0.5 is 24.9 and 29.1 nm. The Brunauer-Emmett-Teller surface area of TM0 and TM0.5 samples is 8.51 and 10.2 m^2^ g^−1^, respectively, suggesting that TM0.5 has a larger specific surface area and greater N_2_ adsorption capability than TM0 sample.

The separation efficiency of photo-generated holes and electrons is confirmed by the transient photocurrent response (I-t curves), as shown in Fig. 3c. TM0.5 sample exhibits higher photocurrent intensity than TM0, which is ascribed to the effective migration of photoelectrons from the conduction band of MoS_2_ to Ti_3_C_2_ nanosheets. The charge carrier recombination/transfer behavior of TM samples is explored by electrochemical impedance spectra (EIS), as presented in Fig. 3d. Among those samples, the biggest and the smallest arc size of Nynquist curve are displayed by TM0 and TM0.5 photocatalysts, respectively, indicating the high conductivity of Ti_3_C_2_ MXene is beneficial to the electron migrate. However, a bigger radius of the arc can be observed in TM2 sample (Fig. S4), which suggests that too high Ti_3_C_2_ loading leads to the increase of carrier transfer impedance. Obviously, the well agreement of I-t and EIS results confirms that the content of Ti_3_C_2_ can affect the transfer of photogenerated carriers.

Figure S5 shows the FT-IR spectrum of TM0 and TM0.5 samples. The absorption bands at 600, 910, 1100, and 1630 cm^−1^ are correspondence to the Mo-S, S-S, Mo-O, and -OH stretching, respectively [51]. The band at about 3350 cm^−1^ is attached to -CH_2_ group from surface water stretching vibration [52]. Compared with TM0 sample, all the peaks of TM0.5 samples exhibit a slight shift, suggesting strong interaction is emerged between MoS_2_ and Ti_3_C_2_ nanosheets.

**Fig. 4:**
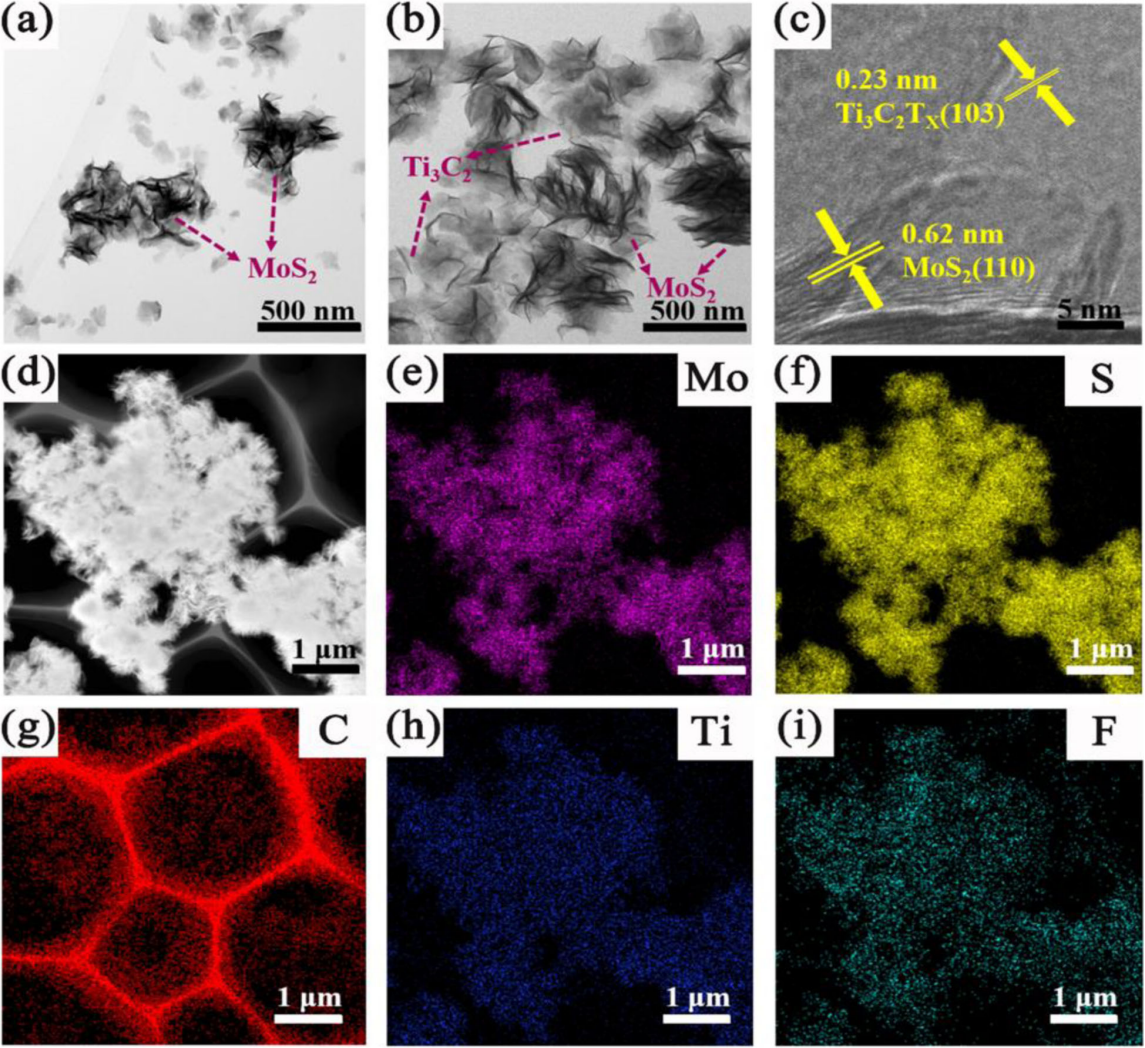
**a**, **b** TEM images of TM0 and TM0.5 samples. **c** HRTEM image of Ti_3_C_2_/MoS_2_. **d** A STEM image. **e**, **f**, **d**, **h**, **i** EDS mapping images of Mo, S, C, Ti, and F elements of TM0.5 sample

HRTEM images of TM0 and TM0.5 composites are further observed in Fig. 4a, b. Overall, the degree of overlap for MoS_2_ nanosheets and agglomeration for MoS_2_ microsphere decreases with Ti_3_C_2_ addition increasing. In detail, for the pure MoS_2_ nanosheets, the overlap for the MoS_2_ can be noticed, which is not beneficial for the absorption of visible light, as shown in Fig. 4a. With the increase of Ti_3_C_2_ addition, the morphology of MoS_2_ gradually changes from crouching to stretching state (Fig. 4b), which could bring out the enlarged specific surface area and increased active sites. The ultrathin layered Ti_3_C_2_ nanosheets are well dispersed in solution and closely contact with MoS_2_. This is favorable for facilitating MoS_2_ nanosheets stretch through strong physical coupling, which will play an important role in electron transfer in photocatalytic process. While, as Ti_3_C_2_ content further increases to 1 and 2 wt%, a large number of MoS_2_ nanosheets randomly overlapping and agglomerating on Ti_3_C_2_ substrates, as shown in Fig. S6a, b.

Figure 4c gives the heterojunction structure of TM0.5. The lattice spacing of 0.23 and 0.62 nm is assigned to (103) crystal plane of Ti_3_C_2_ and (110) crystal plane of MoS_2_, respectively [24, 47]. The intimate-contact heterojunction promotes the transfer and separation of photogenerated carriers and holes at the heterojunction interface [43]. More details of heterojunction structure in TM samples can be observed in Fig. S6c, d. The scanning transmission electron microscopy (STEM) of TM0.5 is displayed in Fig. 4d, and the corresponding EDS mapping of Mo, S, C, Ti, and F is given in Fig. 4e-i. The atomic ratios (Fig. S3) of C, Ti, Mo, and S elements are 62.68, 3.79, 10.56, and 22.97%, respectively. The clear outline of flower-like MoS_2_ grafted on ultra-thin Ti_3_C_2_ nanosheets proves that Ti_3_C_2_ nanosheets coupled with MoS_2_ construct intimate heterojunction successfully. All the evidences of SEM and TEM images indicate that the TM composites are synthesized successfully.

**Fig. 5:**
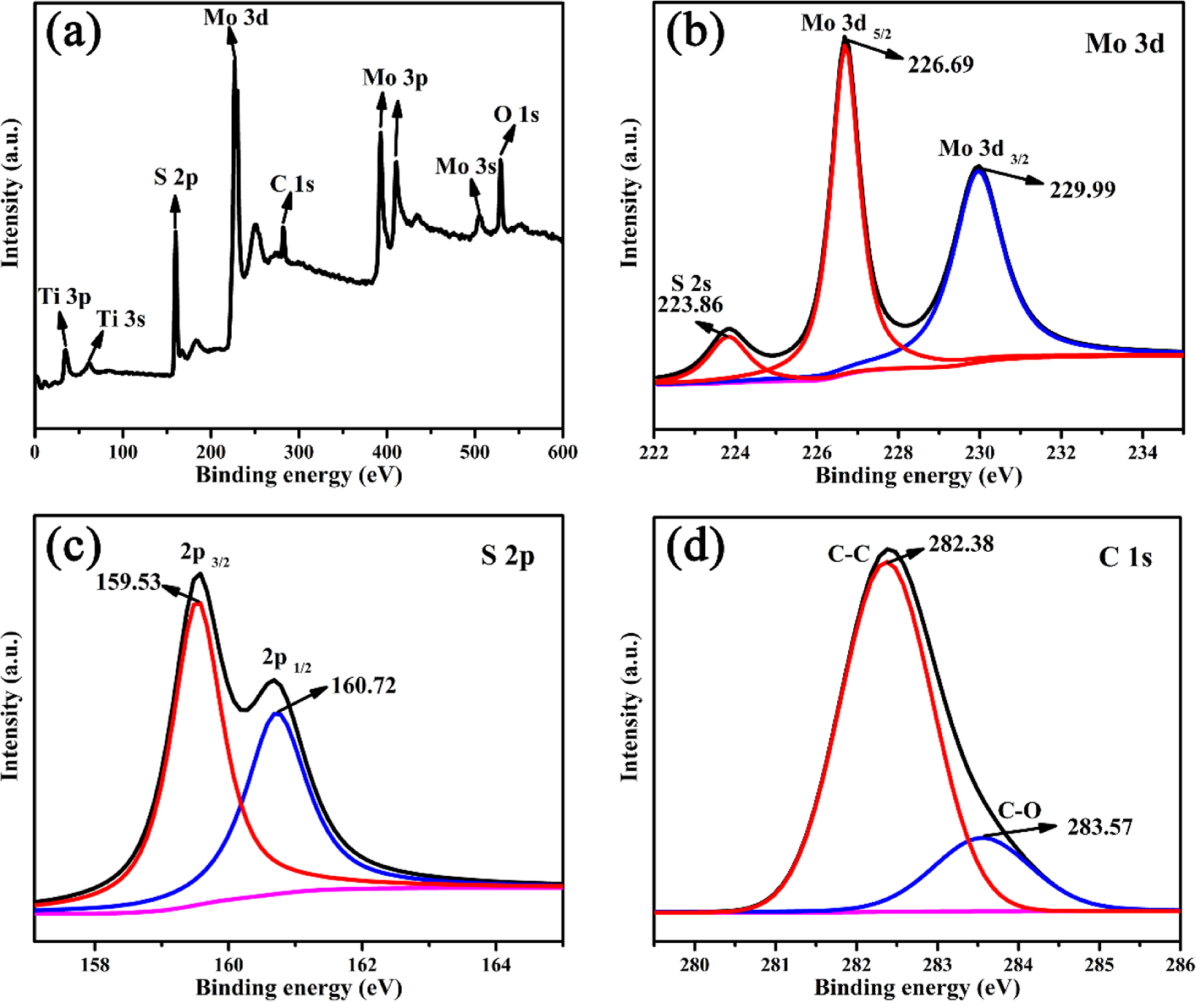
**a** XPS survey spectra and high resolution XPS spectra of **b** Mo 3d, **c** S 2p, **d** C 1 s in TM sample

For further confirming the coexistence of Ti_3_C_2_ and MoS_2_ in the composite, XPS is taken for analyzing the surface chemical composition and states of TM0.5 sample, as shown in Fig. 5. All elements (Mo, S, Ti, O, C) are observed in the XPS survey spectra. Characteristic peaks 36.4, 160.6, 226.8, 283.6, and 529.7 eV are indexed as Ti 3p, S 2p, Mo 3d, C 1 s, and O 1 s, respectively [19]. In Fig. 5b, three peaks at the binding energies of 223.86, 226.69, and 229.99 eV are assigned to S 2 s, Mo 3d_5/2_, and Mo 3d_3/2_, respectively, revealing the existence of Mo^3+^ in TM hybrids. As shown in Fig. 5c, two peaks are situated at 159.53 and 160.72 eV, in accordance with S 2p. The peaks of C 1 s belong to Ti_3_C_2_ is appeared at the binding energies of 282.38 and 283.57 eV, as displayed in Fig. 5d.

**Fig. 6:**
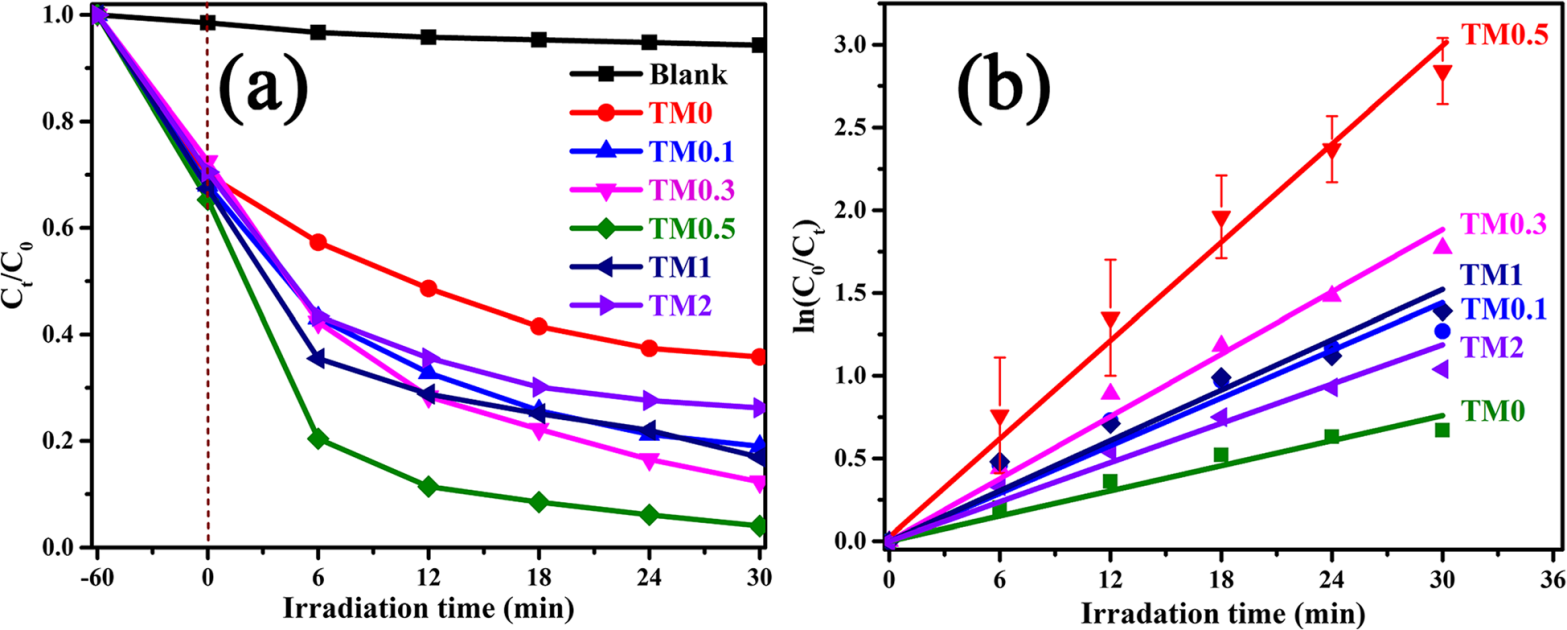
**a** Photocatalytic degradation performance. **b** The corresponding rate constant *k* values of TM0, TM0.1, TM0.3, TM0.5, TM1 and TM2 composites under visible irradiation (30 mg/L MO solution)

Figure 6a, b exhibits the photocatalytic activity for the degradation of MO over various TM samples under visible light irradiation. The blank experiment proves that there is no obvious change in the MO solution within 90 min reaction in the absence of catalyst, as given in Fig. 6a. It turns out that MO molecules are proved to be chemically stable and difficult to be decomposed. The adsorption effect is eliminated before photocatalytic degradation by stirring the mixtures in the dark for 1 h. After being treated in the dark for 60 min, 37~51% of MO is adsorbed by different TM composites. All of the samples demonstrate strong physical adsorption abilities and the TM0.5 sample shows great adsorption ability than others due to the increased specific surface area. After adsorption, subsequent photocatalytic degradation experiments are carried out with equilibrium MO concentration as initial concentration.

Obviously, all the TM composites display higher photodegradation abilities than pristine MoS_2_ under visible light irradiation, suggesting that a small amount of Ti_3_C_2_ MXene addition can enhance the photocatalytic activity of MoS_2_. When the increase of MXene addition from 0 to 0.5 wt%, the total degradation of MO increases dramatically. The highest photocatalytic performance is obtained by TM0.5 sample and 97.4% MO solution is degraded within 30 min. By further increasing the Ti_3_C_2_ addition to 2 wt%, the degradation ability of TM composites catalysts is decreased. This phenomenon can be attributed to the fact that too much Ti_3_C_2_ hinders the absorption of visible light by MoS_2_ nanosheets, reducing photocatalytic activity [53]. The comparison of different TiO_2_-based composites for photocatalytic degradation of MO under visible light irradiation is shown in Table S1.

Moreover, the degradation kinetics of MO have been fitted as plotted according to pseudo-first-order kinetics theory (ln (C_0_/C_t_)) = kt, where *k* is the apparent first-order rate constant, as shown in Fig. 6b. It can be obtained that the kinetics rates constant for TM0, TM0.1, TM0.3, TM0.5, TM1, and TM2 are 0.00135, 0.00308, 0.00454, 0.00836, 0.00401, and 0.0028 min^−1^, respectively. The optimal value of *k* belongs to TM0.5 sample, which is about 6.2 times higher than the TM0.

In order to investigate the photocatalytic activity of TM0.5 composites under various MO concentrations, the degradation for 20, 30, and 50 mg/L of MO solution is given in Fig. S7a. In general, the degradation efficiency of TM0.5 sample decreases as the concentration of MO solution increases. As can be noticed, > 90% of lower concentration MO solution is degraded within 25 min. Figure S7b, c shows the changes of ultraviolet absorption spectra of 30 and 50 mg/L MO solution, respectively. The strong absorption peak of MO solution at 554 nm decreases gradually due to the photodegradation effect of TM0.5. Moreover, TM0.5 sample also exhibits strong degradation ability (nearly 80%) for the degradation of MO (50 mg/L) in 125 min. Above results prove that TM photocatalysts have potential prospects for the degradation of high concentration organic pollutants.

**Fig. 7:**
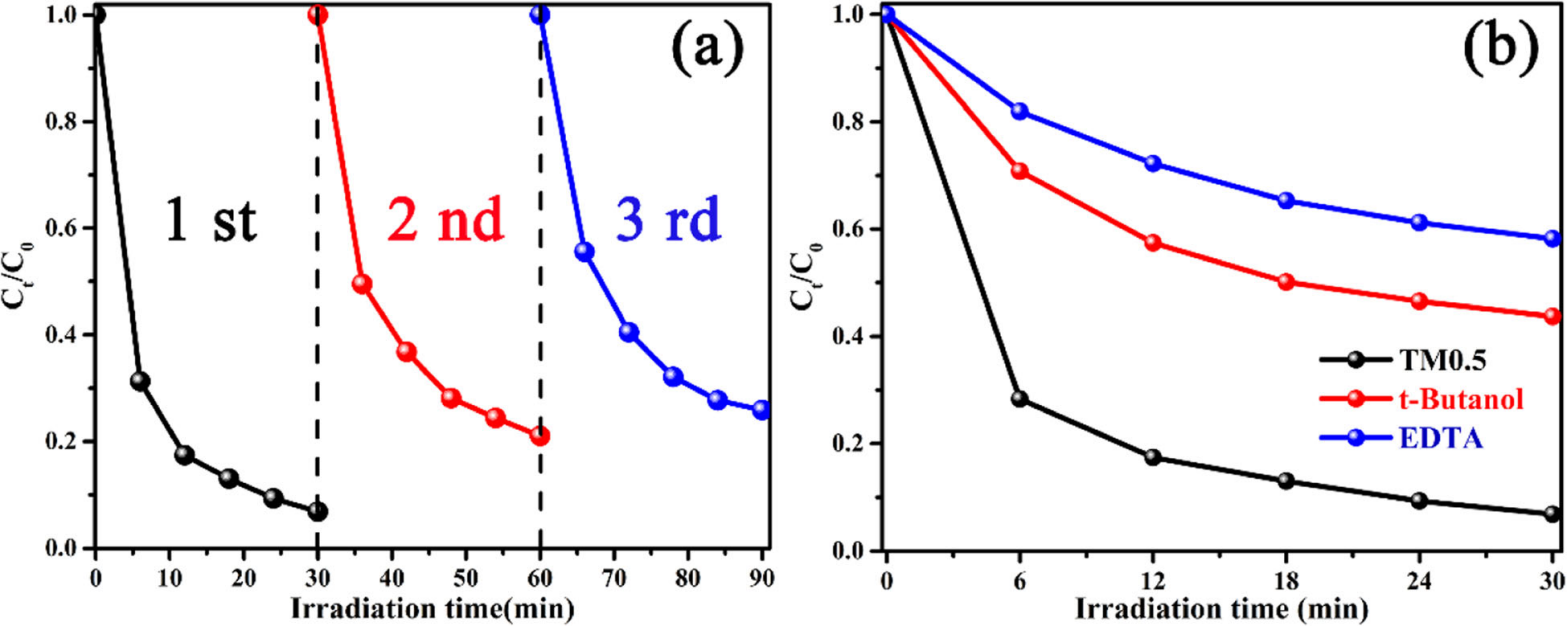
**a** Recycling photocatalytic experiments of TM0.5 sample for photocatalytic degradation of MO by repeating three times under same condition. **b** Effects of different scavengers on the MO photodegradation process under visible light

The stability of photocatalyst is tested by repeating three times under the same condition. Separation of TM0.5 from mixture solution by high-speed centrifugal treatment. The stability of TM samples is revealed in Fig. 7a, the photocatalytic activity of the TM0.5 sample does not decline significantly after 3 recycles of the photodegradation process, which demonstrates that the photocatalyst possesses superior stability and sustainability [54]. The structural stability of photocatalysts is obtained by comparing the XRD before and after use, as shown in Fig. S8.

The potential mechanism of photocatalytic degradation is obtained by trapping experiments. The photogenerated holes (h^+^) and hydroxyl radicals (•OH) play crucial roles in photocatalytic degradation process [21]. Triethanolamine (EDTA) and t-Butanol are introduced as the scavengers to quench active holes (h^+^) and hydroxyl radicals (•OH) under visible light irradiation, respectively. As displayed in Fig. 7b, the TM0.5 composite exhibits the best photocatalytic activity when no scavenger is added. In the presence of EDTA or t-Butanol, the degradation of MO is remarkably inhibited, suggesting that the photogenerated holes and hydroxyl radicals all take part in the photocatalytic reaction. After adding EDTA, the degradation of MO decreases significantly (less than 40%), indicating that holes play a key role in the degradation reaction. Therefore, the principal active species of photocatalytic degradation are photogenerated holes (h^+^), followed by hydroxyl radicals (•OH).

**Fig. 8:**
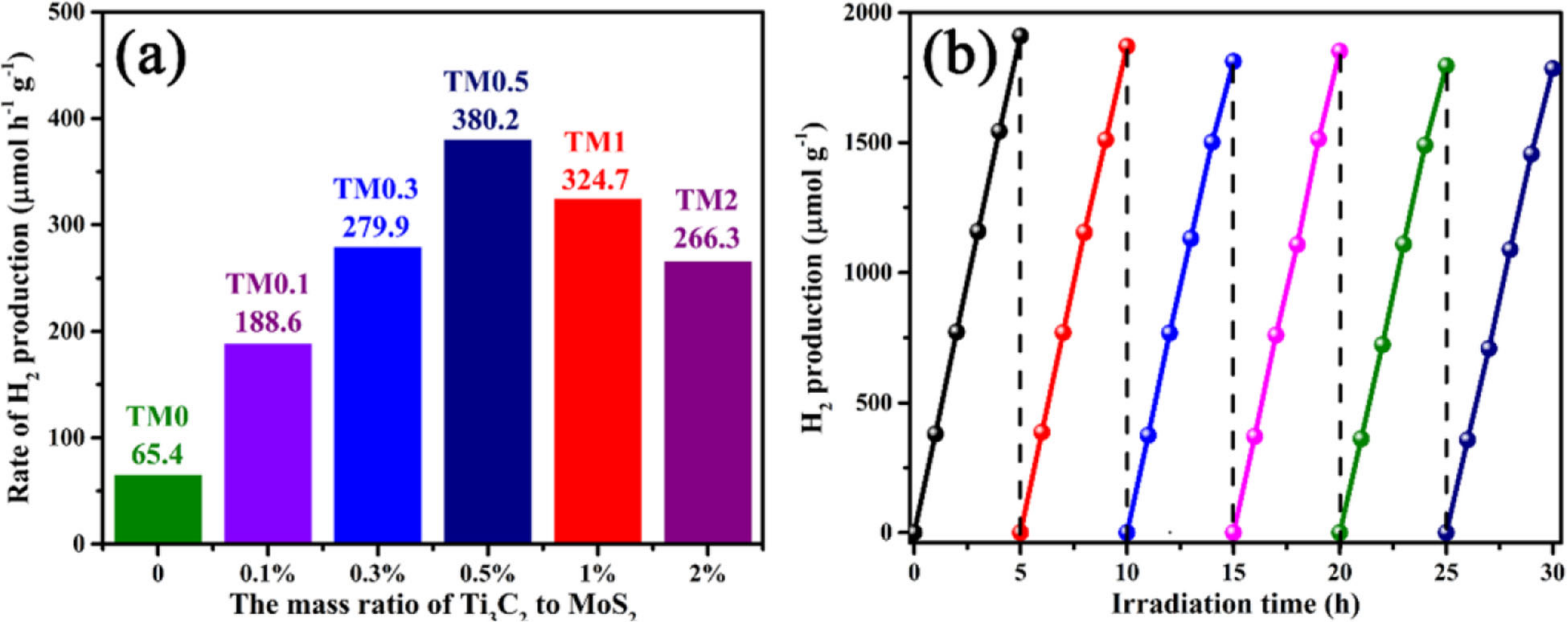
**a** The photocatalytic hydrogen evolution rate of TM0, TM0.1, TM0.3, TM0.5, TM1 and TM2 samples under visible light irradiation. **b** The recycling tests of TM0.5 for water splitting process

The 2D/2D heterojunction of R-scheme Ti_3_C_2_ MXene/MoS_2_ is beneficial to the migration and aggregation of electrons from conduction band of MoS_2_ to the active sites of Ti_3_C_2_, thus accelerating the photocatalytic hydrogen evolution process. Figure 8a presents a comparison of H_2_ production activities with different TM samples under visible light irradiation. The pure MoS_2_ (TM0) sample shows a poor photocatalytic hydrogen production rate (65.4 μmol h^−1^ g^−1^) due to the rapid recombination of photocarrier. The rates of photocatalytic H_2_ production are significantly increased after coupling with Ti_3_C_2_ nanosheets, indicating that the electron acceptors of 2D Ti_3_C_2_ MXene can effectively enhance the electron mobility. The optimal loading of Ti_3_C_2_ in Ti_3_C_2_ MXene/MoS_2_ composites is 0.5 wt%, in accordance with the H_2_ production rate of 380.2 μmol h^−1^ g^−1^. However, the rates of hydrogen production increase with Ti_3_C_2_ loading up to 0.5 wt% and then decrease at a higher Ti_3_C_2_ loading. The hydrogen production rates of TM1 and TM2 samples are 324.7 and 266.3 μmol h^−1^ g^−1^, respectively. The reduction of hydrogen evolution rates at higher Ti_3_C_2_ loading can be described as the excessive Ti_3_C_2_ MXene shielding MoS_2_ from the visible light.

Furthermore, the recoverability of TM0.5 photocatalyst is further analyzed by cyclic photocatalytic hydrogen production tests. As depicted in Fig. 8b, the H_2_ production remains stable after 6 cycles with 5 h intermittence reaction under irradiation, which suggests that Ti_3_C_2_/MoS_2_ composites have strong stability.

**Fig. 9:**
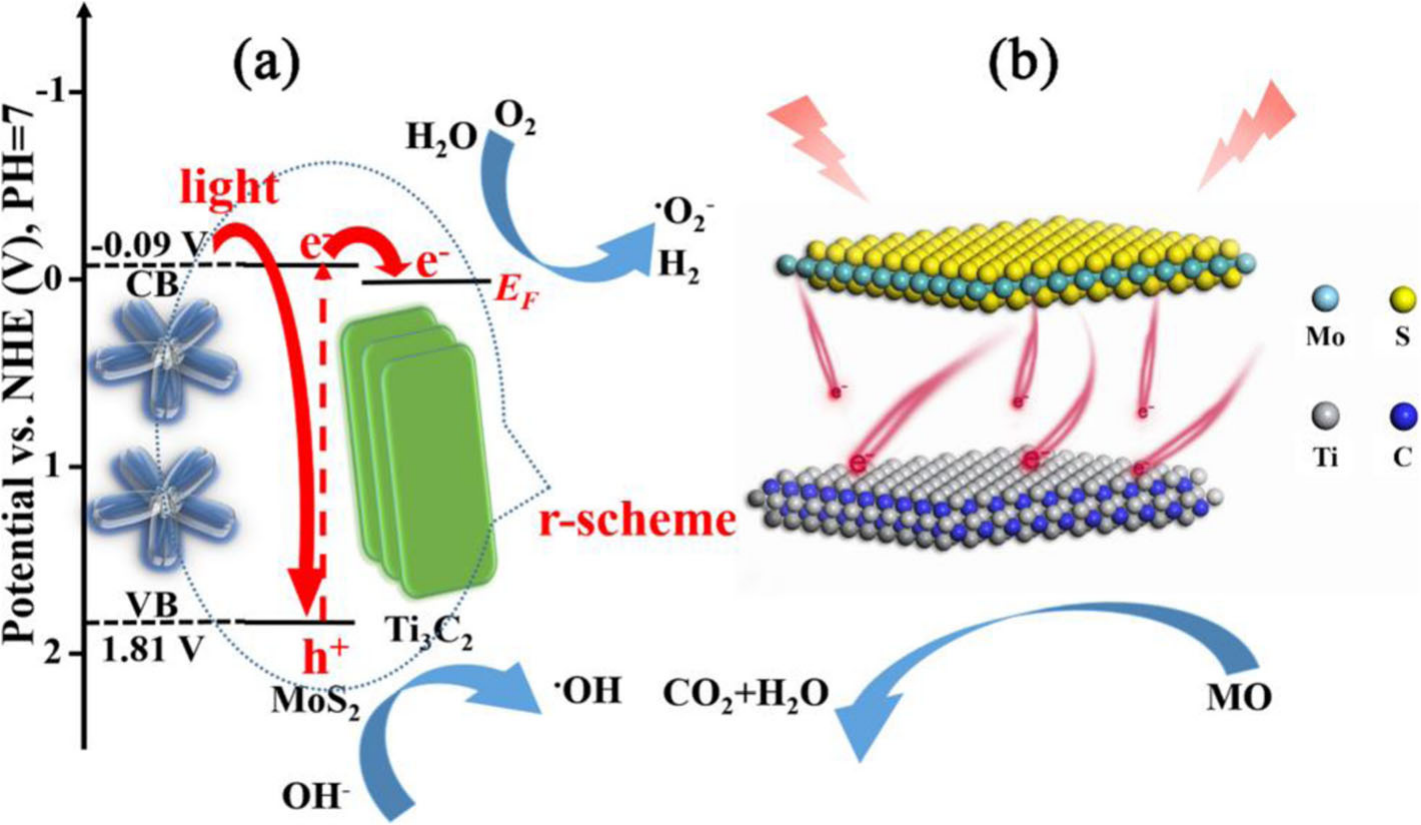
**a** Energy level structure diagram of MoS_2_ and Ti_3_C_2_. **b** Schematic illustration of photo-induced electron transfer process at the heterojunction interface

The probable mechanism of photocatalytic reaction over 2D/2D heterojunction of R-scheme Ti_3_C_2_ MXene/MoS_2_ can be demonstrated in Fig. 9a. The photo-induced electrons arise from the VB of MoS_2_ and transfer to the corresponding CB under visible irradiation. Photoelectrons can transfer quickly from conduction band (CB) of MoS_2_ to Ti_3_C_2_ by close-contact heterojunction due to the greater activeness of the E_F_ of Ti_3_C_2_ than the CB potential of MoS_2_ [55]. In a typical degradation process, a large number of electrons accumulated on the surface of Ti_3_C_2_ MXene reacted with oxygen (O_2_) to produce superoxide radicals (•O_2_^−^). Meanwhile, the hydroxyl ions (OH^−^) and water adsorbed onto the catalyst surface reacted with photogenerated holes to generate hydroxyl radicals (•OH) [46].

The steps of photocatalytic H_2_ evolution reaction are depicted by Eq. (1)-(3) on the active rites of Ti_3_C_2_:

1 $$ {\mathrm{H}}_3{\mathrm{O}}^{+}+{\mathrm{e}}^{-}+\ast \to \mathrm{H}\ast +{\mathrm{H}}_2\mathrm{O} $$

2 $$ {\mathrm{H}}_3{\mathrm{O}}^{+}+{\mathrm{e}}^{-}+\mathrm{H}\ast \to {\mathrm{H}}_2+{\mathrm{H}}_2\mathrm{O} $$

3 $$ \mathrm{H}\ast +\mathrm{H}\ast \to {\mathrm{H}}_2 $$

The active sites can be represented by * in HER process. The surface terminations of Ti_3_C_2_ MXene absorb H_3_O^+^ ion and electron to form an H atom, which is called Volmer reaction, as presented in Eq. (1). The H atom combines with an electron from Ti_3_C_2_ and another H_3_O^+^ to form a hydrogen molecule, which is known as the Heyrovsky mechanism, as depicted in Eq. (2). A H_2_ molecule is formed by two H atoms on the active sites, which is called the Tafel mechanism, as displayed in Eq. (3) [44].

The 2D/2D heterojunction of TM samples is illustrated in Fig. 9b. The photogenerated electrons can rapidly migrate from MoS_2_ to the surface of Ti_3_C_2_ nanosheets due to the electronic transfer channel of 2D/2D heterojunction. The excellent electronic conductivity of 2D Ti_3_C_2_ can effectively extend the separation time and reduce the recombination of photogenerated electron hole pair [56]. Therefore, the photocatalytic activity is enhanced obviously.

## Conclusions

In summary, 2D/2D heterojunction of R-scheme Ti_3_C_2_ MXene/MoS_2_ composites is successfully synthesized by hydrothermal method. The Ti_3_C_2_ MXene/MoS_2_ photocatalysts display remarkably enhanced photocatalytic activity for the degradation of MO and H_2_ evolution reaction compared with pristine MoS_2_. The 0.5 wt% Ti_3_C_2_ MXene/MoS_2_ sample reaches an optimum MO degradation of 97.4% after 30 min irradiation and hydrogen evolution rate of 380.2 μmol h^−1^ g^−1^ under visible irradiation. The morphology and structure analysis confirm that MoS_2_ nanosheets are induced by ultrathin Ti_3_C_2_ MXene from crouching to stretching, which may greatly increase the specific surface area and enhance the light absorption ability. More importantly, Ti_3_C_2_ MXene coupled with MoS_2_ nanosheets can effectively receive and transfer electrons from excited semiconductor, which is beneficial to suppress the charge recombination and improve the interface charge transfer processes. In this work, the constructed novel 2D/2D heterojunction of R-scheme Ti_3_C_2_ MXene/MoS_2_ demonstrates that Ti_3_C_2_ MXene can become a promising cocatalyst in photocatalytic reaction.

## Supplementary information

**Additional file 1: Figure S1.** The XRD of raw Ti_3_AlC_2_ and Ti_3_C_2_. **Figure S2.** (a-d) shows EDS mapping of Mo, Ti and C elements of TM sample; (e) EDS analysis of TM0.5. **Figure S3.** The EDX analysis of TM0.5 sample. **Figure S4.** EIS spectra of TM0, TM0.5 and TM2 powders. **Figure S5.** FT-IR spectra of TM0 and TM0.5. **Figure S6.** TEM (a-b) and HRTEM (c-d) images of TM1 and TM2 samples. **Figure S7.** (a) Comparison on photocatalytic performance of TM0.5 with various concentration of MO solution (20/30/50 mg/L); (b-c) temporal UV-Vis absorption spectra of 30 and 50 mg/L MO solutions after being illuminated by visible light in the presence of TM0.5 sample, respectively. **Figure S8.** The XRD patterns of used and fresh TM0.5 sample. **Table S1.** Different TiO_2_-based composites for photocatalytic degradation of MO under visible light irradiation.

## Data Availability

All data generated or analyzed during this study are included in this published article and its supplementary information files.

## Abbreviations

XRD: X-ray diffraction
SIBs: Sodium-ion batteries
HER: Hydrogen evolution reaction
TM: Ti_3_C_2_ MXene/MoS_2_
FESEM: Field emission scanning electron microscopy
EDS: Energy-dispersive spectrometry
HRTEM: High resolution transmission electron microscopy
FTIR: Fourier transform infrared spectroscopy
DRS: UV-Vis diffuse reflectance spectroscopy
XPS: X-ray photoelectron spectroscopy
EIS: Electrochemical impedance spectroscopy
STEM: Scanning transmission electron microscopy
EDTA: Triethanolamine
