# Supplementary material for: 2D/2D Heterojunction of R-scheme Ti3C2 MXene/MoS2 Nanosheets for Enhanced Photocatalytic Performance
Source: Nanoscale Res Lett. 2020 Apr 9;15:78. doi: 10.1186/s11671-020-03314-z (PMC7145887; doi:10.1186/s11671-020-03314-z)
Supplement: Supplementary file 1 — Additional file 1: Figure S1. The XRD of raw Ti3AlC2 and Ti3C2. Figure S2. (a-d) shows EDS mapping of Mo, Ti and C elements of TM sample; (e) EDS analysis of TM0.5. Figure S3. The EDX analysis of TM0.5 sample. Figure S4. EIS spectra of TM0, TM0.5 and TM2 powders. Figure S5. FT-IR spectra of TM0 and TM0.5. Figure S6. TEM (a-b) and HRTEM (c-d) images of TM1 and TM2 samples. Figure S7. (a) Comparison on photocatalytic performance of TM0.5 with various concentration of MO solution (20/30/50 mg/L); (b-c) temporal UV-Vis absorption spectra of 30 and 50 mg/L MO solutions after being illuminated by visible light in the presence of TM0.5 sample, respectively. Figure S8. The XRD patterns of used and fresh TM0.5 sample. Table S1. Different TiO2-based composites for photocatalytic degradation of MO under visible light irradiation. [file 11671_2020_3314_MOESM1_ESM.docx]

2D/2D Heterojunction of r-scheme Ti_3_C_2_ MXene/MoS_2_ Nanosheets for Enhanced Photocatalytic Performance

**
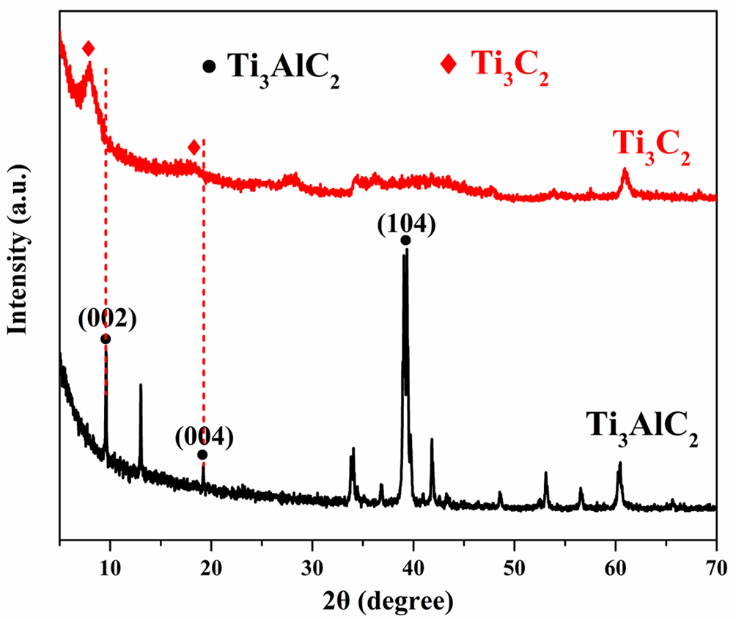
**

**Fig. S1** The XRD of raw Ti_3_AlC_2_ and Ti_3_C_2_.


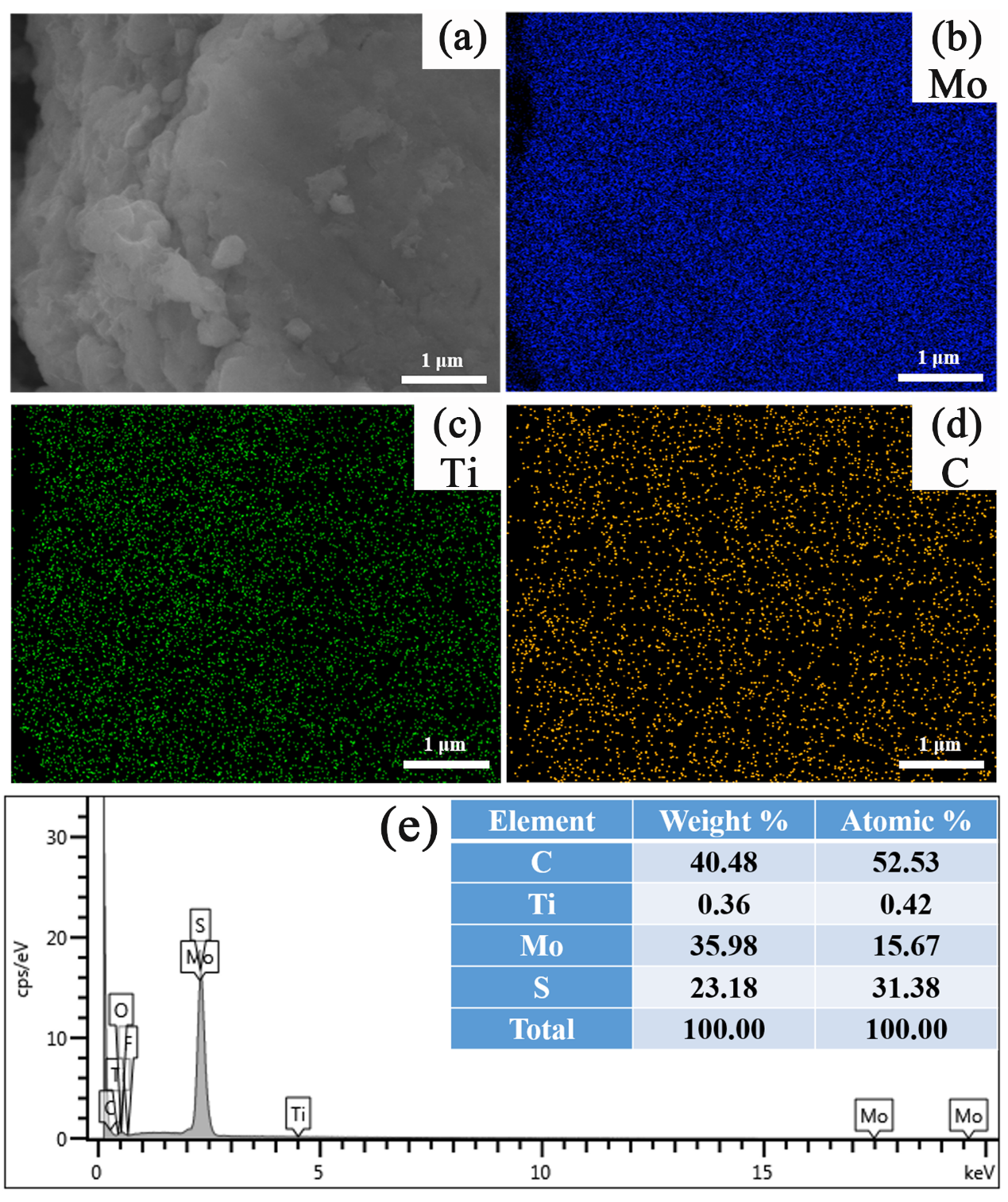


**Fig. S2** (a-d) shows EDS mapping of Mo, Ti and C elements of TM sample; (e) EDS analysis of TM0.5.


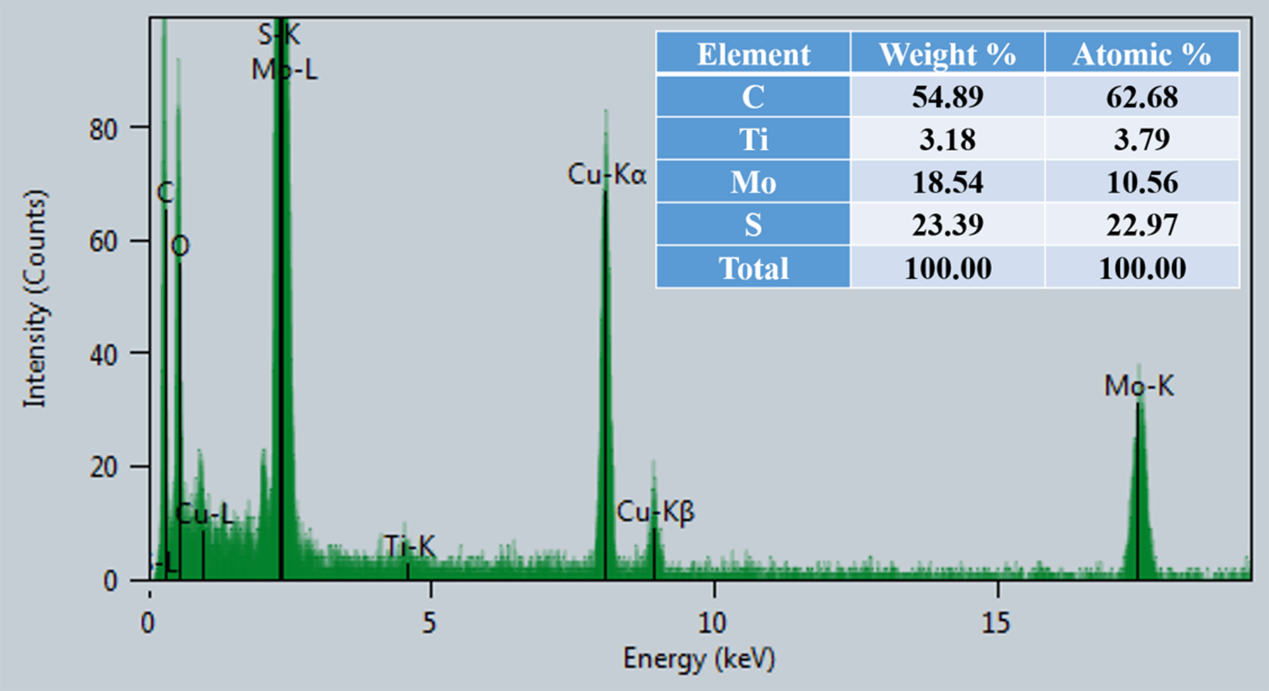


**Fig. S3** The EDX analysis of TM0.5 sample.


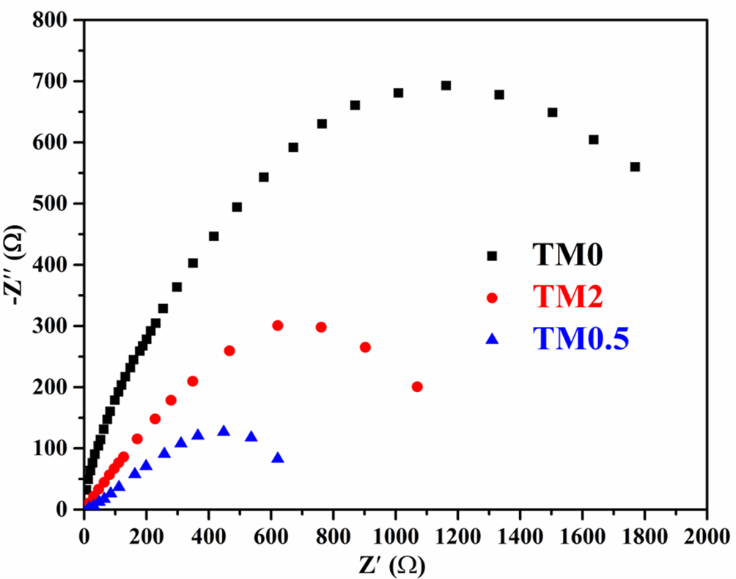


**Fig. S4** EIS spectra of TM0, TM0.5 and TM2 powders.


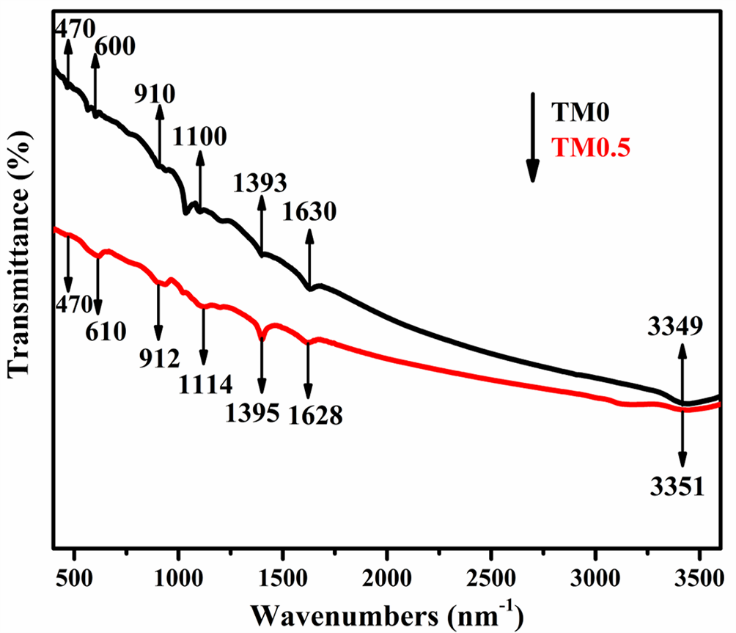


**Fig. S5** FT-IR spectra of TM0 and TM0.5.


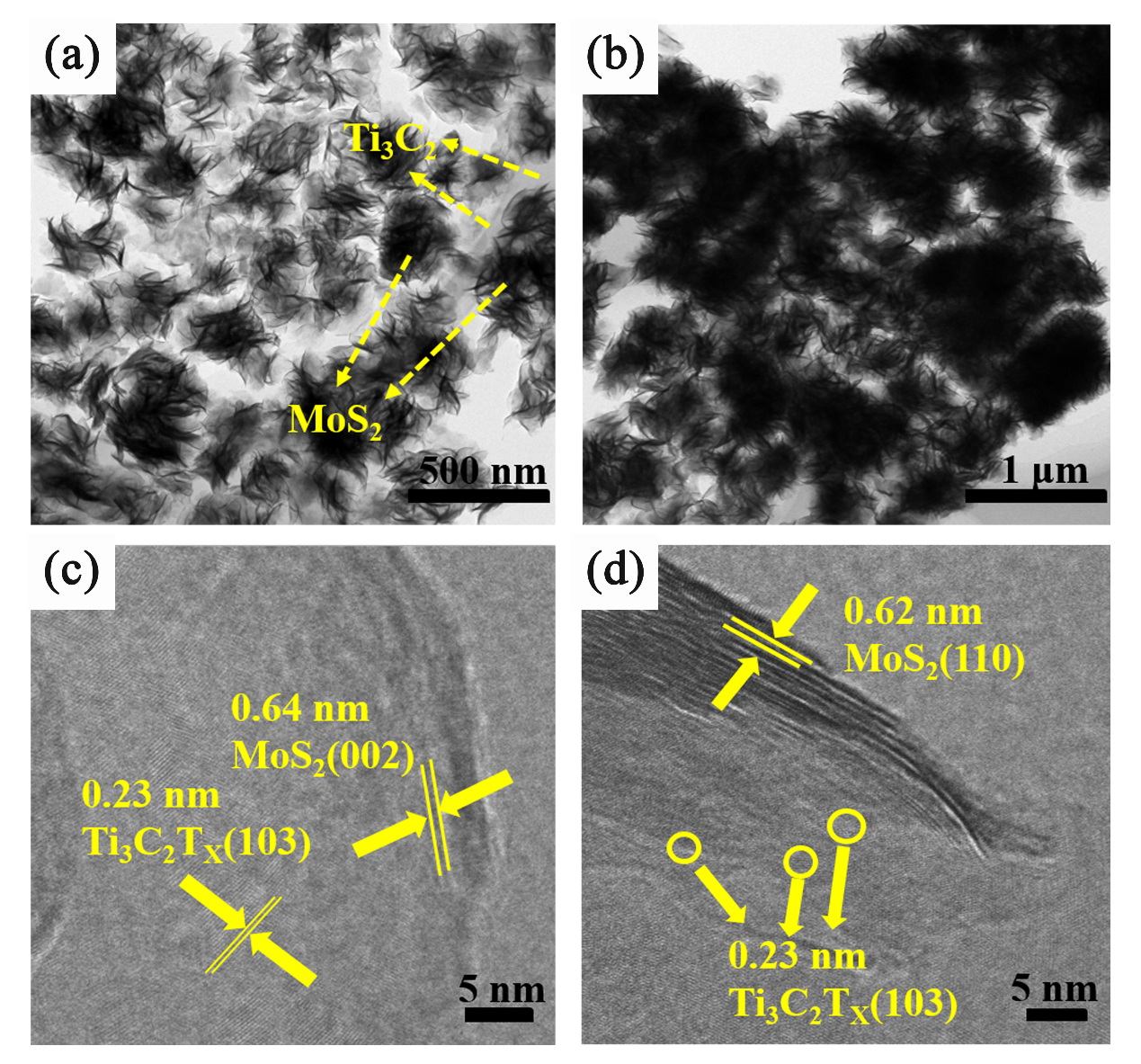


**Fig. S6.** TEM (a-b) and HRTEM (c-d) images of TM1 and TM2 samples.


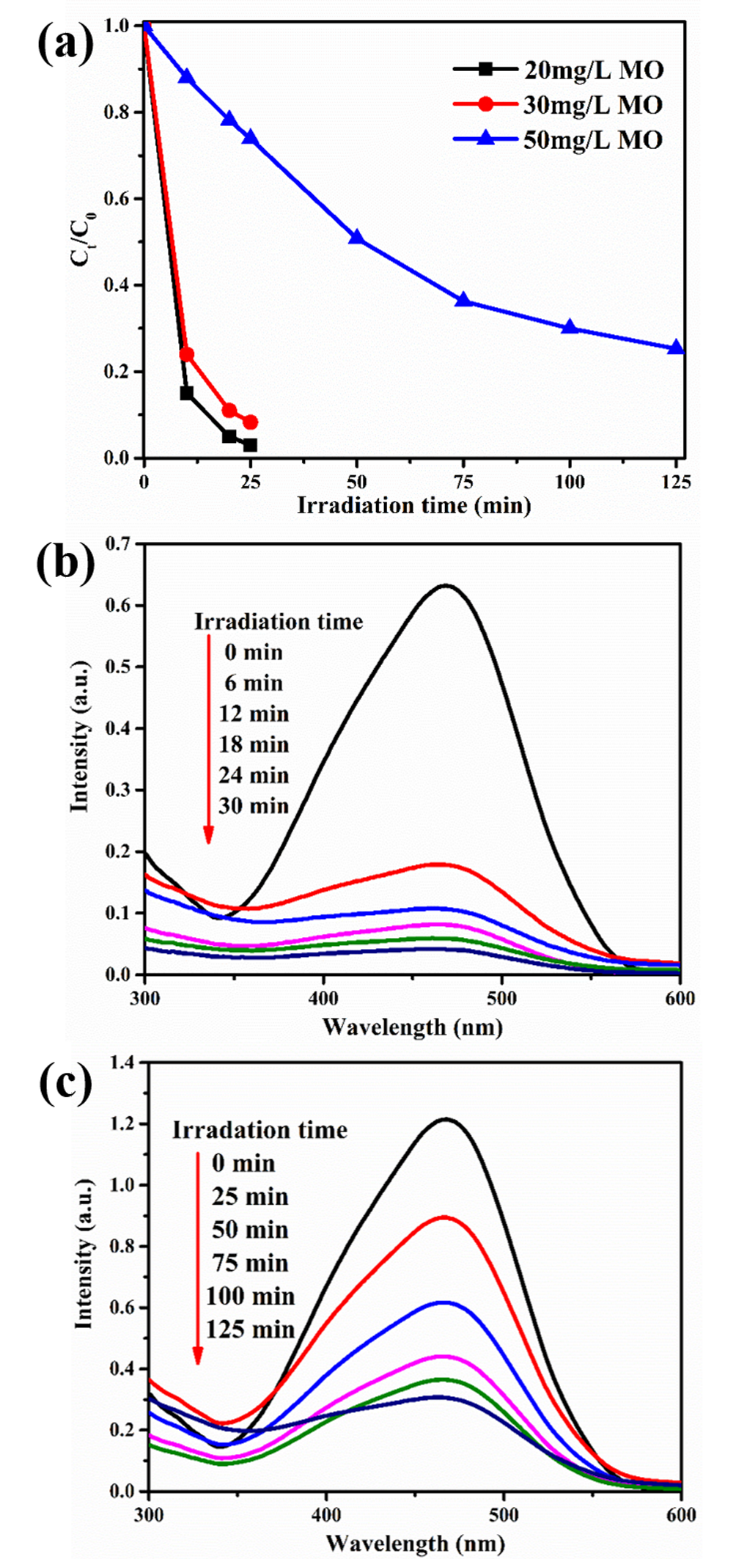


**Fig. S7.** (a) Comparison on photocatalytic performance of TM0.5 with various concentration of MO solution (20/30/50 mg/L); (b-c) temporal UV-Vis absorption spectra of 30 and 50 mg/L MO solutions after being illuminated by visible light in the presence of TM0.5 sample, respectively.


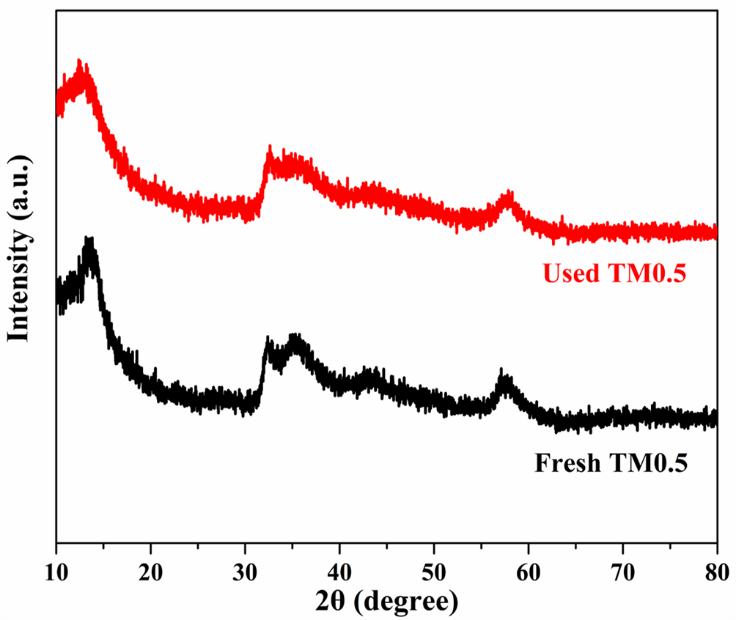


**Fig. S8.** The XRD patterns of used and fresh TM0.5 sample.

**Table.S1** Different TiO_2_-based composites for photocatalytic degradation of MO under visible light irradiation.

| Composite | Visible light source | Catalyst mass | Dye concentration | Degradation efficiency | Refs. |
| --- | --- | --- | --- | --- | --- |
| (Fe, N, B)-TiO_2_ | 1000 W  tungsten halogen lamp | 70 mg | MO, (20 mg/L), 50 mL | 300 min, 73% | [1] |
| Pt-fullerene/TiO_2_ | 8 W halogen lamp | 50 mg | MO, (3.3 mg/L), 50 mL | 120 min, 52% | [2] |
| br-TiO_2_/g-C_3_N_4_ | 300 W Xe lamp | 100 mg | MO, (10 mg/L), 100 mL | 180 min, 55% | [3] |
| TiO_2_-Sn-La | 150 W Xe lamp | 80 mg | MO, (5 mg/L), 50 mL | 120 min, 99% | [4] |
| PMo12/TiO_2_/Ag | 300 W Xe lamp | 20 mg | MO, (20 mg/L), 20 mL | 120 min, 99% | [5] |
| L-Histidine (C, N codoped)-TiO_2_-CdS | 50 W LED arrays | 300 mg | MO, (10 mg/L), 200 mL | 120 min, 95% | [6] |
| In_2_S_3_/anatase TiO_2_ @Ti_3_C_2_T_x_ | 300 W Xe lamp | 60 mg | MO, (20 mg/L), 100 mL | 60 min, 90% | [7] |
| TiO_2_-graphene | 450 W Xe lamp | 30 mg | MO, (10 mg/L), 50 mL | 180 min, 99% | [8] |
| N-doped rutile TiO_2_ | 300 W Xe lamp | 50 mg | MO, (10 mg/L), 25 mL | 120 min, 92% | [9] |
| Ti_3_C_2_ MXene/MoS_2_ | 400 W metal halide lamp | 50 mg | MO, (30 mg/L), 50 mL | 30 min, 97.4% | This work |

Reference:

[1] M. Xing, Y. Wu, J. Zhang, F. Chen, Effect of synergy on the visible light activity of B, N and Fe co-doped TiO_2_ for the degradation of MO, Nanoscale 2 (2010) 1233-1239.

[2] Z. Meng, L. Zhu, J. Choi, M. Chen, W. Oh, Effect of Pt treated fullerene/TiO_2_ on the photocatalytic degradation of MO under visible light, J. Mater. Chem. 21 (2011) 7596-7603.

[3] Y. Zang, L. Li, Y. Xu, Y. Zuo, G. Li, Hybridization of brookite TiO_2_ with g-C_3_N_4_: a visible-light-driven photocatalyst for As^3+^ oxidation, MO degradation and water splitting for hydrogen evolution, J. Mater. Chem. A 2 (2014) 15774-15780.

[4] N. N. Ilkhechi, M. R. Akbarpour, R. Yavari, Z. Azar, Sn^4+^ and La^3+^ co doped TiO_2_ nanoparticles and their optical, photocatalytic and antibacterial properties under visible light, J Mater Sci: Mater Electron 28 (2017) 16658-16664.

[5] H. Shi, Y. Yu, Y. Zhang, X. Feng, X. Zhao, H. Tan, S. U. Khan, Y. Li, E. Wang, Polyoxometalate/TiO_2_/Ag composite nanofibers with enhanced photocatalytic performance under visible light, Appl. Catal. B: Environ. 221 (2018) 280-289.

[6] H. Zangeneh, A. A. Zinatizadeh, S. Zinadini, M. Feyzi, E. Rafiee, D. W. Bahnemann, A novel L-Histidine (C, N) codoped-TiO_2_-CdS nanocomposite for efficient visible photo-degradation of recalcitrant compounds from wastewater, J. Hazard. Mater. 369 (2019) 384-397.

[7] H. Wang, Y. Wu, T. Xiao, X. Yuan, G. Zeng, W. Tu, S. Wu, H. Y. Lee, Y. Tan, J. Chew, Formation of quasi-core-shell In_2_S_3_/anatase TiO_2_@metallic Ti_3_C_2_T_x_ hybrids with favorable charge transfer channels for excellent visible-light- photocatalytic performance, Appl. Catal. B: Environ. 233 (2018) 213-225.

[8] Z. Zhao, T. Kou, L. Zhang, S. Zhai, W. Wang, Y. Wang, Dealloying induced N-doping in spindle-like porous rutile TiO_2_ for enhanced visible light photocatalytic activity, Corrosion Sci. 137 (2018) 204-211.

[9] S. Zargari, R. Rahimi, A. Ghaffarinejad, A. Morsali, Enhanced visible light photocurrent response and photodegradation efficiency over TiO_2_-graphene nanocomposite pillared with tin porphyrin, J. Colloid Interface Sci. 466 (2016) 310-321.
